# Longitudinal trajectory analysis of antipsychotic response in patients with schizophrenia: 6-week, randomised, open-label, multicentre clinical trial

**DOI:** 10.1192/bjo.2020.105

**Published:** 2020-10-22

**Authors:** Minhan Dai, Yulu Wu, Yiguo Tang, Weihua Yue, Hao Yan, Yamin Zhang, Liwen Tan, Wei Deng, Qi Chen, Guigang Yang, Tianlan Lu, Lifang Wang, Fude Yang, Fuquan Zhang, Jianli Yang, Keqing Li, Luxian Lv, Qingrong Tan, Hongyan Zhang, Xin Ma, Lingjiang Li, Chuanyue Wang, Xiaohong Ma, Dai Zhang, Hao Yu, Liansheng Zhao, Hongyan Ren, Yingcheng Wang, Xun Hu, Guangya Zhang, Xiaodong Du, Qiang Wang, Tao Li, for the Chinese Antipsychotics Pharmacogenomics Consortium

**Affiliations:** Mental Health Center and Psychiatric Laboratory, West China Hospital of Sichuan University, China; and West China Brain Research Center, West China Hospital of Sichuan University, China; Mental Health Center and Psychiatric Laboratory, West China Hospital of Sichuan University, China; and West China Brain Research Center, West China Hospital of Sichuan University, China; Mental Health Center and Psychiatric Laboratory, West China Hospital of Sichuan University, China; and West China Brain Research Center, West China Hospital of Sichuan University, China; Peking University Sixth Hospital (Institute of Mental Health), China; and National Clinical Research Center for Mental Disorders and Key Laboratory of Mental Health, Ministry of Health (Peking University), China; Peking University Sixth Hospital (Institute of Mental Health), China; and National Clinical Research Center for Mental Disorders and Key Laboratory of Mental Health, Ministry of Health (Peking University), China; Mental Health Center and Psychiatric Laboratory, West China Hospital of Sichuan University, China; West China Brain Research Center, West China Hospital of Sichuan University, China; Second Xiangya Hospital, Central South University, China; Mental Health Center and Psychiatric Laboratory, West China Hospital of Sichuan University, China; and West China Brain Research Center, West China Hospital of Sichuan University, China; Second Xiangya Hospital, Central South University, China; Beijing Anding Hospital, Institute for Brain Disorders, Capital Medical University, China; Peking University Sixth Hospital (Institute of Mental Health), China; and National Clinical Research Center for Mental Disorders and Key Laboratory of Mental Health, Ministry of Health (Peking University), China; Peking University Sixth Hospital (Institute of Mental Health), China; and National Clinical Research Center for Mental Disorders and Key Laboratory of Mental Health, Ministry of Health (Peking University), China; Beijing HuiLongGuan Hospital, China; Wuxi Mental Health Center, Nanjing Medical University, China; Institute of Mental Health, Tianjin Anding Hospital, China; and Tianjin Medical University General Hospital, Tianjin Medical University, China; Hebei Mental Health Center, China; Second Affiliated Hospital of Xinxiang Medical University, China; Department of Psychiatry, Xijing Hospital, Fourth Military Medical University, China; Wuxi Mental Health Center, Nanjing Medical University, China; Beijing Anding Hospital, Institute for Brain Disorders, Capital Medical University, China; Second Xiangya Hospital, Central South University, China; Beijing Anding Hospital, Institute for Brain Disorders, Capital Medical University, Beijing, China; Mental Health Center and Psychiatric Laboratory, West China Hospital of Sichuan University, China; and West China Brain Research Center, West China Hospital of Sichuan University, China; Peking University Sixth Hospital (Institute of Mental Health), China; and National Clinical Research Center for Mental Disorders and Key Laboratory of Mental Health, Ministry of Health (Peking University), China; Department of Psychiatry, Jining Medical University, China; Mental Health Center and Psychiatric Laboratory, West China Hospital of Sichuan University, China; and West China Brain Research Center, West China Hospital of Sichuan University, China; Mental Health Center and Psychiatric Laboratory, West China Hospital of Sichuan University, China; West China Brain Research Center, West China Hospital of Sichuan University, China; Mental Health Center and Psychiatric Laboratory, West China Hospital of Sichuan University, China; and West China Brain Research Center, West China Hospital of Sichuan University, China; West China Brain Research Center, West China Hospital of Sichuan University, China; and Biobank, West China Hospital of Sichuan University, China; Suzhou Psychiatric Hospital, The Affiliated Guangji Hospital of Soochow University, China; Suzhou Psychiatric Hospital, The Affiliated Guangji Hospital of Soochow University, China; Mental Health Center and Psychiatric Laboratory, West China Hospital of Sichuan University, China; and West China Brain Research Center, West China Hospital of Sichuan University, China; Mental Health Center and Psychiatric Laboratory, West China Hospital of Sichuan University, China; and West China Brain Research Center, West China Hospital of Sichuan University, China

**Keywords:** Trajectories, antipsychotic drugs, schizophrenia, treatment response, clinical trial

## Abstract

**Background:**

Understanding the patterns of treatment response is critical for the treatment of patients with schizophrenia; one way to achieve this is through using a longitudinal dynamic process study design.

**Aims:**

This study aims to explore the response trajectory of antipsychotics and compare the treatment responses of seven different antipsychotics over 6 weeks in patients with schizoprenia (trial registration: Chinese Clinical Trials Registry Identifier: ChiCTR-TRC-10000934).

**Method:**

Data were collected from a multicentre, randomised open-label clinical trial. Patients were evaluated with the Positive and Negative Syndrome Scale (PANSS) at baseline and follow-up at weeks 2, 4 and 6. Trajectory groups were classified by the method of *k*-means cluster modelling for longitudinal data. Trajectory analyses were also employed for the seven antipsychotic groups.

**Results:**

The early treatment response trajectories were classified into a high-trajectory group of better responders and a low-trajectory group of worse responders. The results of trajectory analysis showed differences compared with the classification method characterised by a 50% reduction in PANSS scores at week 6. A total of 349 patients were inconsistently grouped by the two methods, with a significant difference in the composition ratio of treatment response groups using these two methods (χ^2^ = 43.37, *P* < 0.001). There was no differential contribution of high- and low trajectories to different drugs (χ^2^ = 12.52, *P* = 0.051); olanzapine and risperidone, which had a larger proportion in the >50% reduction at week 6, performed better than aripiprazole, quetiapine, ziprasidone and perphenazine.

**Conclusions:**

The trajectory analysis of treatment response to schizophrenia revealed two distinct trajectories. Comparing the treatment responses to different antipsychotics through longitudinal analysis may offer a new perspective for evaluating antipsychotics.

## Background

Antipsychotic drugs are currently the mainstay of treatment for schizophrenia. A meta-analysis of the time course of treatment response reported an early response profile for antipsychotic treatment.^1^ However, previous studies have shown that the treatment response to schizophrenia is heterogeneous. Although most patients’ symptoms improve after treatment, approximately a third of patients respond poorly and are considered to be drug resistant.^2,3^ Some studies have even found that the drugs available to treat schizophrenia are only effective in approximately 50% of patients.^4^ A poor response to treatment may lead to worse community functioning,^5^ symptom exacerbation,^6^ relapse,^7^ reduced patient adherence,^8^ increased risk of admission to hospital, and heavy social and economic burden.^9^ In particular, in the early stages of treatment with antipsychotic drugs, symptom improvement is not stable; therefore, longitudinal profiles of the early efficacy of different antipsychotics can provide novel insight into their different characteristics.

## Importance of understanding response patterns to medication and use of machine learning

Understanding antipsychotic response patterns is critical for successful treatment. The identification of individual response trajectories and potential risk factors associated with poor response trajectories can be a guide for personalised treatments and effective interventions. Previous studies have shown that trajectory analysis may be a useful strategy to reduce heterogeneity in treatment for schizophrenia and may provide novel insight into clinically meaningful patient subgroups.^10^ Some studies with small sample sizes have used parametric analyses and discerned four or more trajectories, but some trajectories merely account for 2% of the sample or even less.^11–16^

When using model-based procedures, the tasks are clustered by data without *a priori* hypotheses. Thus, this approach would provide a better way to avoid issues concerning model selection. *K*-means for longitudinal data (Kml) is an approach related to the partitional classification/cluster analysis of machine learning, which may be an appropriate measure to address model selection issues.^17^ The *k*-means algorithm uses variance distance or dissimilarity measures to identify and classify trajectories, which, as a non-parametric classification method, does not require any assumption regarding normality or parametric assumptions within clusters or the shape of the trajectory and can help to fit the data more closely.^17,18^ Various examples of the implementation of this method, including estimating developmental trajectories of hyperactivity/inattention symptoms, can be found in several studies.^19–21^ However, to our knowledge, few studies have applied such an objective data-driven Kml approach to identify homogeneous trajectories of treatment response in patients with schizophrenia. Therefore, our current study is the first to evaluate antipsychotic drugs from a longitudinal perspective. Moreover, our study has a larger sample size than previous studies.^11–13,15,16^

## Aims

The aims of our study are (a) to explore the response trajectory of antipsychotic drugs with the unsupervised machine learning Kml method and to describe the subgroup features and baseline characteristics of patients with similar treatment responses and (b) to compare the longitudinal treatment responses for seven different antipsychotics.

## Method

### Participants

The participants were recruited from a multicentre, randomised open-label clinical trial that was designed to compare the treatment effectiveness of seven antipsychotic drugs in patients with first-onset psychosis and in those who relapsed. We recruited 3030 patients with schizophrenia from 32 different clinical settings from 6 July 2010 to 30 November 2011, all of whom were enrolled in the China Antipsychotic Pharmacogenomics Consortium. Details of the inclusion and exclusion criteria are provided in the Supplementary File available at https://doi.org/10.1192/bjo.2020.105 (all supplementary figures and tables mentioned can be found in the Supplementary File). Some studies have already been published using these data.^22,23^

Consensus diagnoses were made by at least two experienced psychiatrists based on unstructured interviews with the patients and patients’ families, as well as the review of patients’ medical records. According to the study protocol, participants were randomly assigned (1:1:1:1:1:½:½) to five atypical antipsychotics (risperidone, olanzapine, quetiapine, aripiprazole and ziprasidone) and two typical antipsychotics (perphenazine and haloperidol) using a random allocation sequence generated by a computer. The random allocation was unmasked only to patients and their psychiatrists but not to the researchers following baseline assessments.

The first 2 weeks of treatment were used for drug titration, and the next 4 weeks were the maintenance period. The upper limit of the dose of antipsychotic drugs had to be higher than the lower limit of any target dose range, as recommended by the International Consensus Study of Antipsychotic Dosing.^24^ Other psychotropic drugs were not allowed to be used as concomitant drugs, except for short-acting benzodiazepines for insomnia as well as lorazepam for agitation and psychotic anxiety. The anticholinergic drug hyoscine (up to 6 mg per day) could be prescribed for extrapyramidal symptoms and the beta-blocker propranolol (up to 80 mg per day) could be prescribed for akathisia.

The study was approved by the institutional review board at each site and was conducted by Good Clinical Practice guidelines and the Helsinki Declaration. Written informed consent to participate was obtained from both the patients and their legal guardians. The trial was registered with the Chinese Clinical Trials number: ChiCTR-TRC-10000934.

### Clinical measurements

Demographic and clinical characteristics were collected by trained researchers at baseline. Illness severity was evaluated every 2 weeks using the Positive and Negative Syndrome Scale (PANSS).^25^ The measurements of clinical manifestations included the total score and the positive symptom subscale, negative symptom subscale and general psychopathology subscale scores.

Side-effects were evaluated using the Barnes Akathisia Scale,^26^ Abnormal Involuntary Movement Scale^27^ and Extrapyramidal Symptom Rating Scale.^28^ All raters had received systematic training, and high interrater reliability was achieved. Investigators, staff and patients were masked to the treatment assignments. Where no empirical data for dose conversion were available, we assumed the daily defined dose (i.e. the average maintenance dose per day calculated from the dose recommendations in each drug's product information according to the World Health Organization) to be equivalent.^29^ So we collected the drug doses of all participants at the end of the second week and converted them into risperidone doses by daily defined dose for comparison.

The per cent change in the PANSS score from baseline was calculated by subtracting each value from baseline, dividing by the baseline score, subtracting 30 (the lowest possible PANSS total score), and finally multiplying by 100.^30^ As the subscale score of some participants at baseline was equal to the lowest score of the subscale, the reduction rate in the PANSS subscale was done by subtracting each value from baseline and dividing by the baseline score.

Data values that were 1.5 × interquartile range (IQR) higher than the third quartile or 1.5 × IQR lower than the first quartile were considered outliers. These values were replaced by values at the 0.01 and 0.99 quantiles. Multiple imputation using the R package MICE was applied to impute missing data.^31^

### Statistical analysis

The chi-squared test or Fisher's exact test was used to compare the differences in the distribution of categorical variables among the seven drug groups. Group differences regarding outcome criteria were additionally tested using the chi-squared test or Fisher's exact test for count data; *z*-test/*t*-test was applied for continuous variables; the Wilcoxon rank-sum test was applied for continuous data if the variables did not conform to a normal distribution. After running Fisher's exact test to compare the seven groups, *post hoc* pairwise comparisons adjusted by the false discovery rate (FDR) were performed to identify the differences between groups.

### Treatment response trajectories

To identify similar trajectories of treatment response (reduction rate) during the 6 weeks, *k*-means for clustering the longitudinal data was used with the R Package Kml.^17,18^ Kml was applied with default settings, allowing *k*-means to run for two to six clusters 20 times each. This non-parametric method classified participants into different trajectories, i.e. homogeneous subgroups with similar treatment response patterns. Briefly, each patient was first assigned arbitrarily to one initial trajectory. Second, the centre (*k*-mean) of each trajectory was calculated, and each participant was reassigned to the closest trajectory. The operation was repeated until convergence was achieved. The process from assignment to convergence was then repeated (20 times in this study) to ensure that the solution was not dependent on the initial assignment. Finally, the best solution was determined by a criterion. The Calinski–Harabasz index was applied in this study. The Calinski–Harabasz index describes the closeness through the intraclass dispersion matrix and the separation of the interclass dispersion matrix. The larger the Calinski–Harabasz index is, the better the clustering result is.

All the statistical analyses described above were performed in R 3.6.1.

## Results

### Participant characteristics

**Fig. 1:**
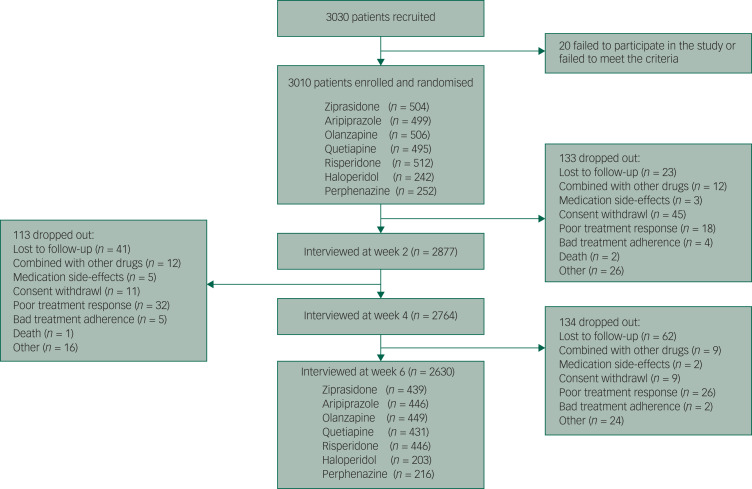
The trial profile.

Of the 3030 patients with schizophrenia, 20 were excluded from further evaluation because of the failure to meet the inclusion criteria or to participate in the study. In total, 2630 participants finished the study. The study sample and the reasons for dropping out are shown in Fig. 1. Participants who dropped out (*n* = 380, 12.62%) tended to be younger and drug-naive and to have higher educational degrees, a shorter duration of illness and milder symptoms. However, there was no difference in the type of medication between the patients who completed the study and those who dropped out (the details are shown in Supplementary Table 1). The baseline demographic characteristics of the patients treated with the seven antipsychotic drugs are shown in Supplementary Table 2. The pattern of missing data is shown in Supplementary Fig. 1, and multiple imputation was applied to impute missing data. There was no significant difference in the data after imputation (*P* > 0.05) (Supplementary Fig. 2).

### Trajectories of treatment response

**Fig. 2:**
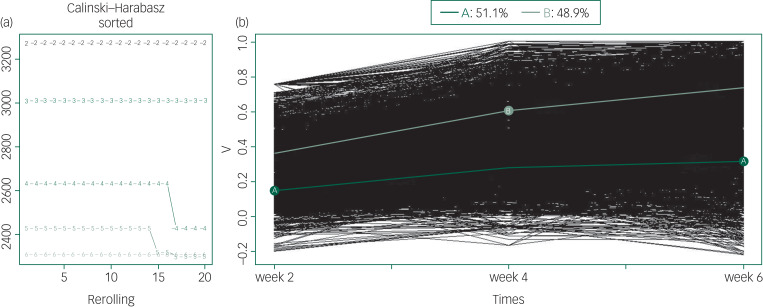
Kml trajectory analysis of treatment response (all patients). (a) The optimal number of clusters to separate patients into groups with homogeneous treatment response over time. The *x*-axis represents the number of runs for two to six clusters. The *y*-axis represents the Calinski–Harabasz index. The curve marked with the number 2 represents the two-trajectory solution and explains the data best as it has the highest Calinski–Harabasz index. (b) The treatment trajectory of all patients. The *y*-axis represents the reduction rate of PANSS scale. Light green line (B) corresponds to a high trajectory or better treatment response (48.9% of patients) and dark green line (A) corresponds to the low trajectory or worse treatment response (51.1% of patients). The thin lines (black) represent individual patient profiles.

In the trajectory analyses of treatment response, values of fit criteria improved steadily from a six-trajectory solution to a two-trajectory solution. The Calinski–Harabasz index of two-trajectory solution was the largest (see Supplementary Table 3). According to the fit criterion, we yielded a two-trajectory solution as the best model (Fig. 2).

**Fig. 3:**
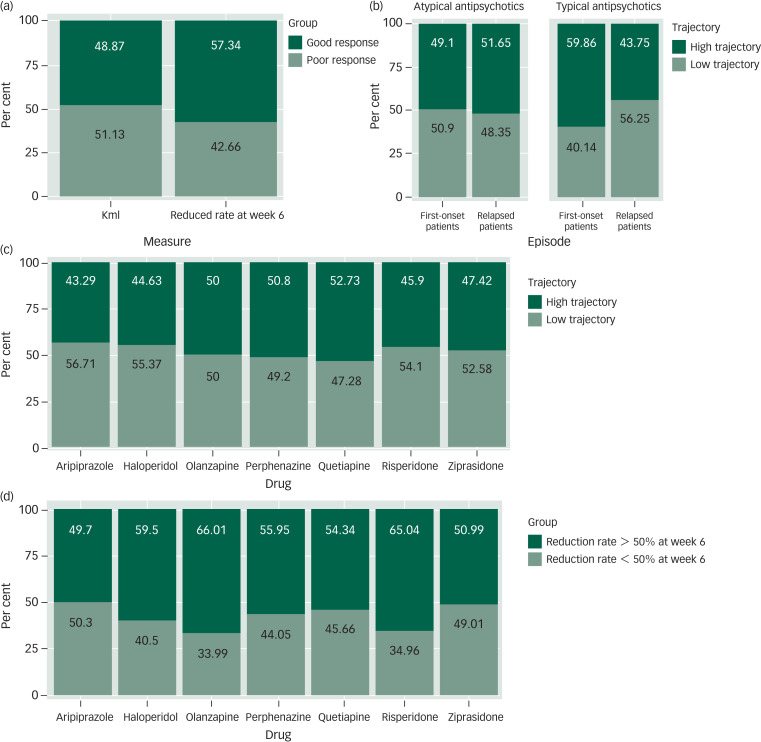
Comparison of findings using the trajectory analysis and dichomtomous threshold methods. (a) The proportion of patients using trajectory analysis and dichotomous thresholds methods for a good response (dark green) versus a poor response (light green). (b) The proportion of patients in a first-episode and patients who relapsed by trajectories (high or low). Data for patients treated with atypical antipsychotics and typical antipsychotics are shown separately. (c) The proportion of patients taking each of the seven antipsychotic drugs by trajectories (high or low). (d) The proportion of patients taking each of seven antipsychotic drugs by dichotomous thresholds methods. Reduction rate at week 6 >50% or <50%. Percentage figures are indicated within bars.

In this pattern, 1471 (48.9% of the patients who entered the study) participants were assigned to the high-trajectory group, and they responded better to treatment. The average rates of change in PANSS total score in the high-trajectory group were 35.84%, 60.51%, and 73.58% at weeks 2, 4 and 6, respectively. The low-trajectory group included 1539 (51.1% of the patients who entered the study) patients. The average rates of change in the PANSS total score were 15.16%, 28.10%, and 31.99% at weeks 2, 4 and 6, respectively. The treatment response of the low-trajectory group was also poorer in terms of positive symptoms, negative symptoms and general symptoms (see details in Supplementary Table 4 and Fig. 3). There was a significant difference in the reduction rate of the PANSS total score and subscale scores between patients in the high- and low-trajectory groups (Table 1, Supplementary Table 4 and Figs 3 and 4). In addition, we performed the same analysis on patients in a first episode, and the results were similar (Supplementary Fig. 5).

**Table 1:** Comparisons of demographics and baseline characteristics of patients separated into high- and low trajectories (all patients)

Demographic	High trajectory (*n* = 1471)	Low trajectory (*n* = 1539)	*P*
Age, years: mean (s.d.)	31.28 (8.29)	31.56 (8.23)	0.361
Gender male, *n* (%)	718 (48.8)	754 (49.0)	0.228
First-onset psychosis, *n* (%)	444 (30.2)	421 (27.4)	0.094
Education, *n* (%)			0.002
Doctor	2 (0.1)	1 (0.1)	
Master	8 (0.5)	5 (0.3)	
Bachelor	108 (7.3)	135 (8.8)	
College	134 (9.1)	166 (10.8)	
High school	350 (23.8)	428 (27.8)	
Middle school	578 (39.3)	576 (37.4)	
Primary school	269 (18.3)	217 (14.1)	
Illiterate	22 (1.5)	11 (0.7)	
Body mass index (kg/m^2^), mean (s.d.)	21.87 (5.78)	21.90 (7.92)	0.919
Age of onset, years: mean (s.d.)	25.84 (7.26)	24.71 (6.62)	<0.001
Illness duration, months: mean (s.d.)	66.45 (66.63)	84.11 (73.10)	<0.001
Family history, *n* (%)	303 (20.6)	338 (22.0)	0.385
Doses,^a^ mg: mean (s.d.)	7.17 (3.03)	7.44 (2.83)	0.011
PANSS Total, mean (s.d.)	89.46 (16.11)	89.38 (14.51)	0.888
PANSS% change (week 2), mean (s.d.)	35.84 (16.68)	15.16 (12.48)	<0.001
PANSS% change (week 4), mean (s.d.)	60.51 (15.34)	28.10 (15.31)	<0.001
PANSS% change (week 6), mean (s.d.)	73.58 (13.54)	31.99 (19.62)	<0.001

PANSS, Positive and Negative Syndrome Scale.

a. The drug doses was converted into risperidone doses by daily defined dose.

The baseline characteristics and outcomes of the high- and low-trajectory groups are shown in Table 1. Participants in the high-trajectory group had a later age of onset, a shorter duration of disease and a lower dose of drugs. There were also differences in the distribution of educational degrees between the two groups. There was no significant difference in other baseline characteristics between the two groups. Among the participants in a first-episode, there was no difference in education between the trajectory groups, and the remaining results were very similar (Supplementary Table 5).

### Comparisons between trajectory analysis and the dichotomous thresholds method

Previous studies have suggested that at least a 50% reduction in the PANSS score from baseline is the cut-off value for defining treatment response.^32^ Based on that, we compared the difference between the trajectory analysis and simple dichotomous thresholds (as indentified by the responder) to evaluate the treatment response. A total of 349 patients were inconsistently grouped by the two methods. Forty-seven patients were in the high-trajectory group with the reduction rate in the PANSS score in the sixth week at less than 50%; 302 patients were in the low-trajectory group, but the reduction rate in the sixth week was more than 50% (Supplementary Fig. 6). The average rates of change in the PANSS total score of these four groups is shown in Supplementary Fig. 7.

In addition, there was a significant difference in the composition ratio of treatment response groups using these two methods (χ^2^ = 43.37, *P <* 0.001). The percentage of patients with a >50% reduction in the PANSS score in the sixth week (*n* = 1726, 57.34%) was significantly higher than that of patients in the high-trajectory group (*n* = 1471, 48.87%) (see Fig. 3(a)).

We also compared the baseline characteristics of the treatment response groups by dichotomous threshold methods. The results showed that there were significant differences in the age at first onset, the duration of illness and the level of education between the two groups (*P* < 0.05), which was consistent with the results of the Kml grouping (Supplementary Table 6).

### Comparison of antipsychotic drugs

Longitudinal trajectory analyses were run separately for each antipsychotic drug group. Furthermore, the analyses were performed on those participants in a first episode and participants who had relapsed in each antipsychotic drug group. These results showed that all drug groups could accept a two-trajectory solution as the best model, and the profiles of response to seven antipsychotics were similar (see Supplementary Figs. 8–15).

In the groups of participants with typical antipsychotic drugs, the proportion was different between the first-episode group and the relapse group (χ^2^ = 10.51, *P* = 0.001). More participants were classified into the high-trajectory group in the first-onset group (*n* = 85, 59.9%), whereas more patients were classified into the low-trajectory group among the relapse group (*n* = 154, 43.80%). This pattern was not found in the atypical antipsychotic drug groups (χ^2^ = 1.33, *P* = 0.25) (Supplementary Table 7 and Fig. 3(b)).

Comparing the treatment response trajectories of the seven drugs separately, there was no differential contribution of the composition of the high-trajectory and low-trajectory groups to the different drugs (χ^2^ = 12.52, *P* = 0.051). The results of the analyses are shown in Fig. 3(c) and Supplementary Table 7.

We also compared the difference in the composition ratio of seven drugs grouped by a cut-off value of 50% for the reduction rate at week 6, and the results were significantly different. Olanzapine and risperidone, which had a higher proportion of >50% reduction in the sixth week, performed better than aripiprazole, quetiapine, ziprasidone and perphenazine. The difference was still found after FDR correction (*P*_FDR_ < 0.05) (Supplementary Table 8).

## Discussion

### Treatment response trajectories

In this study, we performed unsupervised machine learning Kml to classify homogeneous clusters of 6-week response trajectories in patients with schizophrenia. The patients in the high-trajectory group (48.9%) responded well to the treatment, as evidenced by the approximately 70% reduction in the PANSS total score in the sixth week. The level of improvement was considered to be the degree of ‘very much improved’ at a global clinical level.^33^ Furthermore, the other group of patients (51.1%) showed an approximately 30% improvement in their symptoms across the course of the 6-week trial, which was only marginally discernible.^33^

The treatment response trajectory in our study is different from the results of previous trajectory analyses.^11,15,16^ Most previous studies used the total score of the scale rather than the reduction rate to analyse the trajectory of treatment response, which may be one of the reasons for the difference in results. However, clinical trials often used the change from baseline scores to evaluate the effects of different interventions, and ‘response’ is defined as a scale score reduction rate that exceeds a specific value.^13^ Thus, the use of the reduction rate of the total PANSS score in our study may be more appropriate. Moreover, our study used the Kml method, which does not require any normality or parametric assumptions within clusters.^18^ This is important in the present case because of the nature of the variable that was clustered, i.e. the reduction rate of the total PANSS score. A previous trajectory analysis used the Kml technique and they found that over 70% of the patients with schizophrenia treated with clozapine and chlorpromazine were assigned to the good treatment response trajectory, whereas this figure was approximately 50% in the refractory group of patients.^10^ However, the small size of the study may have resulted in an overestimation of the proportion of patients expected to have a good treatment response trajectory.

Whether baseline characteristics affect the treatment response has shown contradictory results in previous studies.^34^ Some have been able to come to such a conclusion, finding that gender,^11,35^ age,^11,36^ age at onset^35,37,38^ and illness duration^39^ affect the treatment response. Our study found that participants in the high-trajectory group had a later age at onset and a shorter duration of disease, which is similar to previous studies. The dose of the drug was lower in the high-trajectory group, which may be related to the better response to treatment in this group. The results obtained by trajectory analysis were similar to those obtained by the dichotomous thresholds method, which also increases the credibility of our results. However, the identified trajectory groups may have differed at baseline in other ways that were not assessed in this analysis. For example, genetic testing and imaging examination might help to further differentiate response trajectories within this large, pooled patient population.

### Comparing treatment trajectories among different antipsychotics

In regard to the trajectories of different antipsychotic drugs, all drug groups could be classified into two trajectories. In the results of the longitudinal effect of typical and atypical antipsychotics, typical antipsychotics performed better in the first-onset group than the relapse group, whereas atypical antipsychotics performed equally in the two groups. Previous studies also found that antipsychotic treatment responses were reduced or delayed in the face of relapse.^40^ However, the smaller sample size of typical antipsychotic drugs may also have resulted in the difference.

Our study also proposes a new perspective to evaluate antipsychotic drugs. To date, there have been limited longitudinal studies on antipsychotic drugs. An analysis of two 6-week trials showed that antipsychotic treatment with haloperidol or olanzapine was more likely than placebo to be associated with a trajectory of ‘dramatic response’.^12^ Previously, some trajectory analyses suggested possible differences in treatment responses among different antipsychotic drugs. One study showed that patients taking olanzapine had a higher proportion in the trajectory of patients with the most improvement than patients in the other drug groups.^15^ Another analysis observed that patients in trajectories with clear worsening of symptoms were more likely to have been treated with quetiapine or risperidone and less likely to have been treated with olanzapine or aripiprazole.^11^ However, fewer kinds of drugs were included in these studies, and there was no analysis or comparison of the specific trajectories of each drug. The effect of drugs on a high trajectory might be more stable. Our results suggested that there was no difference in the distribution of seven antipsychotic drugs between the high- and low-trajectory groups by comparing the longitudinal drug effect.

Interestingly, by comparing the treatment response of the seven antipsychotics by dichotomous thresholds methods (comparing the composition ratio of patients whose reduction rate in the PANSS score was more than and less than 50% at the sixth week), the results showed that individuals taking olanzapine and risperidone responded better to treatment, which is significantly different from the results derived from the comparison using trajectory analysis. End-point threshold-based cut-off values, though convenient, may be misleading in terms of the underlying biology. For example, patients with 49% remission and those showing a 51% response would be divided into two groups, even if they belonged to the same treatment response trajectory. The end-point cut-off value does not take the instability of symptoms and the difference in the onset time of different patients into account. It is difficult to distinguish between transient and permanent results. Compared with dichotomous threshold methods, longitudinal analysis allows changes in treatment responses over time, takes into account all available data from each time point, and identifies treatment response groups consisting of patients whose response patterns are sufficiently similar to one another and sufficiently different from patients in other groups in an outcome measure over time.^11,12^ The two methods for evaluating treatment response have a different emphasis. However, our study follow-up consisted of only 6 weeks. These approaches need to be applied in further research with a longer follow-up to validate the current conclusions.

### Strengths and limitations

The current study also has several limitations. First, the trajectory analysis approach is exploratory rather than definitive and requires replication. Second, we focused on the treatment response trajectory only during the first 6 weeks of treatment. Given that schizophrenia is a chronic disease, a longer-term study is suggested as a key point of future studies to validate the current conclusions. Third, patients with a history of treatment resistance were excluded from the trial, which may have obscured the extent of treatment response and potential trajectories with extreme scores. Finally, the dose of each drug was constant. In the real world, the dose of drugs of individual patients varies with the change of symptoms. The constant dose in our study may not reflect the best effect of drugs, so it is necessary to study the treatment response further in a real-world sample. However, our study is the first to compare seven different antipsychotic drugs through their longitudinal effects, and it includes a large sample size that can better represent the population with schizophrenia. In addition, the use of the Kml method does not rely on prespecified hypotheses regarding the final number of clusters or the most reliable thresholds and contains information from several time points. Moreover, trajectory analysis uses PANSS per cent reduction rather than PANSS total scores to better evaluates treatment response.

### Implications

In conclusion, the current study identified that patients with schizophrenia could be divided into two trajectories: a high-trajectory group with a better treatment response and a low-trajectory group with a poorer treatment response. Longitudinal comparisons of treatment response trajectories and dichotomous threshold methods of drug treatment response were significantly different. Therefore, comparing the treatment responses of different antipsychotics through longitudinal analysis may offer a new perspective to evaluate antipsychotic drugs.

## Data Availability

The data are not publicly available due to their containing information that could compromise the privacy of research participants.

## Supplementary material

For supplementary material accompanying this paper visit http://dx.doi.org/10.1192/bjo.2020.105. click here to view supplementary material
