# Longitudinal trajectory analysis of antipsychotic response in patients with schizophrenia: 6-week, randomised, open-label, multicentre clinical trial – CORRIGENDUM

**DOI:** 10.1192/bjo.2021.995

**Published:** 2021-08-20

**Authors:** Minhan Dai, Yulu Wu, Yiguo Tang, Weihua Yue, Hao Yan, Yamin Zhang, Liwen Tan, Wei Deng, Qi Chen, Guigang Yang, Tianlan Lu, Lifang Wang, Fude Yang, Fuquan Zhang, Jianli Yang, Keqing Li, Luxian Lv, Qingrong Tan, Hongyan Zhang, Xin Ma, Lingjiang Li, Chuanyue Wang, Xiaohong Ma, Dai Zhang, Hao Yu, Liansheng Zhao, Hongyan Ren, Yingcheng Wang, Xun Hu, Guangya Zhang, Xiaodong Du, Qiang Wang, Tao Li

**Keywords:** Trajectories, antipsychotic drugs, schizophrenia, treatment response, clinical trial

https://doi.org/10.1192/bjo.2020.105, Published online by Cambridge University Press, 22 August 2020. In the original publication, one of the grant numbers was published incorrectly in the Funding section. This has now been corrected in both the online PDF and HTML versions of this article. The authors apologise for this error.
