# Supplementary material for: Longitudinal trajectory analysis of antipsychotic response in patients with schizophrenia: 6-week, randomised, open-label, multicentre clinical trial
Source: BJPsych Open. 2020 Oct 22;6(6):e126. doi: 10.1192/bjo.2020.105 (PMC7745240; doi:10.1192/bjo.2020.105)
Supplement: Supplementary file 1 [file bjosup.zip › S2056472420001052sup002.docx]

**Longitudinal Trajectory Analysis of Antipsychotic Response in Patients with Schizophrenia：A Six-week, Randomized, Open-Label, Multicenter Clinical Trial**

**Supplementary**

**Contents**

[The Chinese Antipsychotics Pharmacogenomics Consortium members 2](#_Toc51932914)

[Inclusion and exclusion criteria 3](#_Toc51932915)

**Supplementary Table**

[Table S1 4](#_Toc51932916)

[Table S2 5](#_Toc51932917)

[Table S3 7](#_Toc51932918)

[Table S4: 8](#_Toc51932919)

[Table S5 9](#_Toc51932920)

[Table S6 10](#_Toc51932921)

[Table S7 11](#_Toc51932922)

[Table S8 12](#_Toc51932923)

**Supplementary Figure**

[Figure S1 13](#_Toc51932924)

[Figure S2 14](#_Toc51932925)

[Figure S3 15](#_Toc51932926)

[Figure S4 16](#_Toc51932927)

[Figure S5 17](#_Toc51932928)

[Figure S6 18](#_Toc51932929)

[Figure S7 19](#_Toc51932930)

[Figure S8 20](#_Toc51932931)

[Figure S9 21](#_Toc51932932)

[Figure S10 22](#_Toc51932933)

[Figure S11 23](#_Toc51932934)

[Figure S12 24](#_Toc51932935)

[Figure S13 25](#_Toc51932936)

[Figure S14 26](#_Toc51932937)

[Figure S15 27](#_Toc51932938)

# The Chinese Antipsychotics Pharmacogenomics Consortium members

Dai Zhang, MD, PhD; Weihua Yue, MD, PhD; Hao Yan, PhD; Hongyan Zhang, MD, PhD; Yueqin Huang, MD; Jun Yan, MD; Tianmei Si, MD, PhD; Xin Yu, MD, PhD; Tao Li, MD, PhD; Wei Deng, MD; Xun Hu, PhD; Lingjiang Li, MD, PhD; Liwen Tan, MD; Xue Han, MD; Jiansong Zhou, MD; Qi Chen, MD; Chuanyue Wang, MD, PhD, Xin Ma, MD; Guigang Yang, MD; Yunlong Tan, MD; Fude Yang, MD; Yongfeng Yang, MD; Hongxing Zhang, MD; Luxian Lv, MD; Bo Du, MD; Keqing Li, MD; Guangming Xu, MD; Jianli Yang, MD; Cuicui Ma, MD; Chao Jin, MD; Wenbin Ma, MD; Gang Zhang, MD; Wei Wang, MD; Zaohuo Cheng, MD; Guoyang Qi, MD; Xuebing Chen, MD; Honghui Chen, MD; Yunchun Chen, MD; Qiongrong Tan, MD; Rongxin Zhu, MD; Jianxiong Fan, MD; Ning Zhang, MD; Liying Yang, MD; Zhiyong Li, MD; Congpei Zhang, MD; Chuanhua Lu, MD; Lei Su, MD; Jisheng Tang, MD; Yuping Liu, MD; Yuping Ning, MD; Shutao Pang, MD; Guanjun Wang, MD; Shenghai Wang, MD; Xuanyin Huang, MD; Rongke Wang, MD; Zhili Zou, MD; Huaqing Meng, MD; Bin Hu, MD; Lihua Yu, MD; Guangya Liu, MD; Tiansheng Guo, MD; Bo Wang, MD; Xueqin Yu, MD; Ying Sun, MD; Duanfang Cai, MD; Youguo, MD; Ming Luo, MD; Yueliang Zhang, MD; Xiaoping Ge, MD; Yueqing Ding, MD; Jun Li, MD; Haijun Wang, MD; Deping Chen, MD; Fuhua Zeng, MD; Jun He, MD; Yifei Xu, MD; Guangxiang Zheng, MD; Wei Jian, MD; Wenjun Mao, MD; Shiwu Yang, MD; Chenglin Li, MD.

# Inclusion and exclusion criteria

Inclusion criteria: 1) aged between 18 and 45; 2) Chinese Han population; 3) diagnosed with schizophrenia using Structured Clinical Interview for Diagnostic and Statistical Manual of Mental Disorders, fourth edition (DSM-IV) – Patient Version (SCID-P); 4) physically healthy; 5) laboratory tests within normal limits.

Exclusion criteria: 1) received a diagnosis of schizoaffective disorder, mental retardation, or other neurocognitive disorders; 2) had a history of serious adverse reactions to the proposed treatments; 3) had a history of treatment resistance defined by persistent severe symptoms despite adequate trial of one of the proposed treatments or prior treatment with clozapine; 4) were pregnant or breast-feeding; 5) had a serious and unstable medical condition

Table S1: Univariable analysis of factors associated with dropout.

| Demographic | Finished(n=2630) | Dropped out(n=380) | P-value |
| --- | --- | --- | --- |
| Age(years),  mean(SD) | 31.73 (8.23) | 29.32 (8.19) | <0.001 |
| Gender male, n(%) | 1282 (48.7) | 190 (50.0) | 0.687 |
| First-onset |  |  |  |
| Education, n(%) |  |  | 0.019 |
| Doctor | 3 (0.1) | 0 (0.0) |  |
| Master | 11 (0.4) | 2 (0.5) |  |
| Bachelor | 201 (7.6)  253 (9.6) | 42 (11.1)  47 (12.4) |  |
| College |  |  |  |
| High school | 668 (25.4)  1038 (39.5) | 110 (28.9)  116 (30.5) |  |
| Middle school |  |  |  |
| Primary school | 426 (16.2)  30 (1.1) | 60 (15.8)  3 (0.8) |  |
| Illiterate |  |  |  |
| BMI(kg/m2), mean(SD) | 21.91 (7.26)  25.39 (7.00) | 21.74 (4.33)  24.39 (6.58) | 0.658  0.009 |
| Age of onset(years), mean(SD) |  |  |  |
| Illness duration(months),  mean(SD) | 77.29 (70.50) | 62.67 (69.53) | <0.001 |
| Family history | 564 (21.4) | 77 (20.3) | 0.646 |
| Drugs |  |  | 0.415 |
| Aripiprazole | 446 (17.0) | 53 (13.9) |  |
| Olanzapine | 449 (17.1) | 57 (15.0) |  |
| Quetiapine | 431 (16.4) | 64 (16.8) |  |
| Ziprasidone | 439 (16.7) | 65 (17.1) |  |
| Risperidone | 446 (17.0) | 66 (17.4) |  |
| Haloperidol | 203 (7.7) | 39 (10.3) |  |
| Perphenazine | 216 (8.2) | 36 (9.5) |  |
| PANSS Total,  mean (SD) | 25.59 (4.77) | 24.95 (4.12) | 0.013 |
| PANSS Positive,  mean (SD) | 22.02 (6.73) | 20.01 (6.65) | <0.001 |
| PANSS Negative,  mean (SD) | 42.28 (8.52) | 41.21 (7.81) | 0.021 |
| PANSS General,  mean (SD) | 89.89 (15.43) | 86.17 (14.04) | <0.001 |

Table S2 Baseline demographic characteristics among patients treated with seven antipsychotic drugs.

|  | Aripiprazole  n=499 | Olanzapine  n=506 | Quetiapine  n=495 | Ziprasidone  n=504 | Risperidone  n=512 | Haloperidol  n=242 | Perphenazine  n=253 | Overall  n=3010 |
| --- | --- | --- | --- | --- | --- | --- | --- | --- |
| age (mean (SD)) | 31.62 (8.27) | 30.95 (8.28) | 31.32 (8.23) | 30.89 (8.33) | 31.53 (8.19) | 32.53 (8.22) | 31.95 (8.22) | 31.42 (8.25) |
| Gender male, n(%) | 255 (51.1) | 250 (49.4) | 249 (50.3) | 252 (50.0) | 223 (43.6) | 115 (47.5) | 128 (50.8) | 1472 (48.9) |
| Age of onset(years), mean(SD) | 25.35 (6.58) | 24.92 (6.97) | 25.12 (6.96) | 25.00 (6.98) | 25.57 (7.07) | 26.27 (7.25) | 24.97 (7.06) | 25.26 (6.96) |
| Illness duration(months),  mean(SD) | 76.46 (70.88) | 74.86 (68.75) | 75.62 (70.31) | 71.41 (68.96) | 73.25 (69.29) | 77.69 (70.98) | 84.27 (78.53) | 76.65 (70.93) |
| First-onset, n(%) | 137 (27.5) | 145 (28.7) | 138 (27.9) | 148 (29.4) | 155 (30.3) | 74 (30.6) | 68 (27.0) | 865 (28.74) |
| Education, n(%) |  |  |  |  |  |  |  |  |
| Doctor | 0 (0.0) | 1 (0.2) | 0 (0.0) | 1 (0.2) | 1 (0.2) | 0 (0.0) | 0 (0.0) | 3 (0.1) |
| Master | 1 (0.2) | 3 (0.6) | 3 (0.6) | 6 (1.2) | 0 (0.0) | 0 (0.0) | 0 (0.0) | 13 (0.4) |
| Bachelor | 39 (7.8) | 48 (9.5) | 35 (7.1) | 43 (8.5) | 49 (9.6) | 19 (7.9) | 10 (4.0) | 243 (8.1) |
| College | 55 (11.0) | 53 (10.5) | 50 (10.1) | 56 (11.1) | 50 (9.8) | 18 (7.4) | 18 (7.1) | 300 (10.0) |
| High school | 133 (26.7) | 136 (26.9) | 121 (24.4) | 115 (22.8) | 146 (28.5) | 63 (26.0) | 64 (25.4) | 778 (25.9) |
| Middle school | 185 (37.1) | 189 (37.4) | 190 (38.4) | 188 (37.3) | 187 (36.5) | 96 (39.7) | 119 (47.2) | 1154 (38.3) |
| Primary school | 83 (16.6) | 72 (14.2) | 92 (18.6) | 88 (17.5) | 71 (13.9) | 43 (17.8) | 37 (14.7) | 486 (16.1) |
| Illiterate | 3 (0.6) | 4 (0.8) | 4 (0.8) | 7 (1.4) | 8 (1.6) | 3 (1.2) | 4 (1.6) | 33 (1.1) |
| BMI(kg/m2), mean(SD) | 21.78 (6.09) | 21.52 (6.11) | 22.57 (10.99) | 21.91 (5.21) | 21.71 (6.61) | 21.30 (5.85) | 22.33 (3.98) | 21.88 (6.96) |
| Family history, n(%) | 110 (22.0) | 108 (21.3) | 99 (20.0) | 103 (20.4) | 113 (22.1) | 54 (22.3) | 54 (21.4) | 641(21.3) |
| PANSS Positive,  mean (SD) | 25.50 (4.37) | 25.17 (4.66) | 26.01 (4.70) | 25.38 (4.94) | 25.20 (4.69) | 25.75 (4.66) | 25.87 (4.89) | 25.51 (4.70) |
| PANSS Negative,  mean (SD) | 21.52 (6.84) | 21.58 (6.56) | 21.85 (6.59) | 22.15 (6.77) | 21.32 (6.89) | 21.74 (6.97) | 22.65 (6.66) | 21.77 (6.75) |
| PANSS General,  mean (SD) | 42.48 (8.54) | 41.01 (8.42) | 42.84 (8.12) | 42.38 (8.37) | 42.03 (8.37) | 42.08 (8.40) | 42.17 (9.06) | 42.14 (8.44) |
| PANSS Total,  mean (SD) | 89.50 (15.45) | 87.76 (15.51) | 90.71 (14.73) | 89.91 (15.31) | 88.56 (15.11) | 89.56 (15.18) | 90.69 (16.02) | 89.42 (15.31) |

Table S3: Results of kml for all patients

|  | 2 | 3 | 4 | 5 | 6 |
| --- | --- | --- | --- | --- | --- |
| Calinski Harabatz | 3274.171 | 3010.083 | 2633.977 | 2432.812 | 2313.246 |
| Proportion per trajectory(%) |  |  |  |  |  |
| Trajectory 1 | 51.13 | 44.22 | 33.39 | 31.40 | 27.54 |
| Trajectory 2 | 48.87 | 28.87 | 30.47 | 30.07 | 21.89 |
| Trajectory 3 |  | 26.91 | 19.23 | 16.88 | 21.73 |
| Trajectory 4 |  |  | 16.91 | 15.88 | 11.79 |
| Trajectory 5 |  |  |  | 5.78 | 11.73 |
| Trajectory 6 |  |  |  |  | 5.32 |

Table S4: Comparisons of mean change rates of PANSS scales between high and low trajectories

| Mean change rates of PANSS | High trajectory  (n=1471) | Low trajectory  （n=1539） | P-value |
| --- | --- | --- | --- |
| PANSS% positive change(week2), mean (SD) | -28.34 (15.13) | -14.55 (12.31) | <0.001 |
| PANSS% positive change(week4), mean (SD)) | 48.55 (13.52) | 27.96 (15.27) | <0.001 |
| PANSS% positive change(week6), mean (SD) | 58.68 (12.44) | 31.74 (19.36) | <0.001 |
| PANSS% negative change(week2), mean (SD) | 18.17 (15.18) | 5.97 (11.04) | <0.001 |
| PANSS% negative change(week4), mean (SD) | 31.38 (17.44) | 13.24 (16.17) | <0.001 |
| PANSS% negative change(week6), mean (SD) | 39.40 (18,45) | 15.49 (17.15) | <0.001 |
| PANSS% general change(week2), mean (SD) | 22.02 (11.76) | 9.20 (10.11) | <0.001 |
| PANSS% general change(week4), mean (SD) | 35.49 (11.80) | 16.91 (12.79) | <0.001 |
| PANSS% general change(week6), mean (SD) | 43.11 (11.61) | 19.19 (14.57) | <0.001 |
| PANSS% total change(week2), mean (SD) | 35.84 (16.68) | 15.16 (12.48) | <0.001 |
| PANSS% total change(week4), mean (SD | 60.51 (15.34) | 28.10 (15.31) | <0.001 |
| PANSS% total change(week6), mean (SD) | 73.58 (13.54) | 31.99 (13.62) | <0.001 |

Table S5: Comparisons of demographics and baseline characteristics of patients separated into high and low trajectories (first-onset patients)

| Demographic | High trajectory  (n=430) | Low trajectory  （n=435） | P-value |
| --- | --- | --- | --- |
| Age(years),  mean(SD) | 29.07 (8.29) | 28.83 (8.11) | 0.67 |
| Gender male, n(%) | 222 (51.6) | 198 (45.5) | 0.084 |
| Education, n(%) |  |  | 0.424 |
| Doctor | 1 ( 0.2) | 0 ( 0.0) |  |
| Master | 2 ( 0.5) | 1 ( 0.2) |  |
| Bachelor | 36 ( 8.4) | 36 ( 8.3) |  |
| College | 41 ( 9.5) | 59 (13.6) |  |
| High school | 100 (23.3) | 108 (24.8) |  |
| Middle school | 164 (38.1) | 162 (37.2) |  |
| Primary school | 79 (18.4) | 65 (14.9) |  |
| Illiterate | 7 ( 1.6) | 4 ( 0.9) |  |
| BMI(kg/m2), mean(SD) | 21.02 (4.62) | 21.61 (11.93) | 0.34 |
| Age of onset(years), mean(SD) | 27.44 (7.82) | 26.13 (7.00) | 0.01 |
| Illness duration(years),  mean(SD) | 1.63 (2.80) | 2.71 (3.98) | <0.001 |
| Family history, n(%) | 86 (20.0) | 87 (20.0) | 1 |
| PANSS Total,  mean (SD) | 88.98 (16.82) | 88.18 (14.15) | 0.449 |

Table S6: Comparisons of demographics and baseline characteristics of patients separated by dichotomous thresholds methods (all patients)

| Demographic | Reduced rate at week6 > 50%  (n=1682) | Reduced rate at week6 ≤ 50%  （n=1328） | P-value |
| --- | --- | --- | --- |
| Age(years),  mean(SD) | 31.34 (8.22) | 31.56 (8.23) | 0.52 |
| Gender male, n(%) | 819 (48.69) | 653 (49.17) | 0.79 |
| First-onset,n(%) | 497 (29.55) | 368 (27.71) | 0.27 |
| Education, n(%) |  |  | <0.001 |
| Doctor | 2 (0.1) | 1 (0.1) |  |
| Master | 9 (0.5) | 4 (0.3) |  |
| Bachelor | 122 (7.3) | 121 (9.1) |  |
| College | 153 (9.1) | 147 (11.1) |  |
| High school | 402 (23.9) | 376 (28.3) |  |
| Middle school | 664 (39.5) | 490 (36.9) |  |
| Primary school | 304 (18.1) | 182 (13.7) |  |
| Illiterate | 26 (1.5) | 7 (0.5) |  |
| BMI(kg/m2), mean(SD) | 21.94 (5.65) | 21.82 (8.33) | 0.64 |
| Age of onset(years), mean(SD) | 25.69 (7.13) | 24.72 (6.69) | <0.001 |
| Illness duration(months),  mean(SD) | 70.43 (69.31) | 84.53 (72.19) | <0.001 |
| Family history, n(%) | 352 (20.9) | 289 (21.8) | 0.59 |
| PANSS Total,  mean (SD) | 89.36 (16.02) | 89.50 (14.36) | 0.80 |
| PANSS% change(week2), mean (SD) | 32.35 (17.54) | 16.30 (14.05) | <0.001 |
| PANSS% change(week4), mean (SD) | 56.05 (17.94) | 28.60 (17.28) | <0.001 |

Table S7 Comparisons of the trajectories of the first-episode patients and relapsed patients with atypical and typical antipsychotics

| drugs | first onset High Trajectory(％) | first onset Low Trajectory(％) | relapse High Trajectory(％) | relapse Low Trajectory(％) | χ^2^ | p-value |
| --- | --- | --- | --- | --- | --- | --- |
| atypical | 355(49.1%) | 368(50.9%) | 926(51.6%) | 867(48.4%) | 1.33 | 0.25 |
| typical | 85(59.9%) | 57(40.1%) | 154(43.8%) | 198(56.2%) | 10.51 | 0.001 |

Table S8 Odds ratio (95％ confidence interval) of pairwise comparison of composition ratio between groups.

|  | Odds ratio of composition ratio between groups with reduced rate of PANSS higher than 50％ and lower than 50％ at week 6 | | | | | | |
| --- | --- | --- | --- | --- | --- | --- | --- |
| Odds ratio of composition ratio between high and low trajectory groups | aripiprazole | 1.96  (1.51-2.56)* | 1.2  (0.93-1.56) | 1.12  (0.87-1.45) | 1.88  (1.45-2.44)* | 1.49  (1.08-2.06) | 1.29  (0.94-1.76) |
|  | 1.31  (1.01-1.69) | olanzapine | 0.61  (0.47-0.8)* | 0.57  (0.44-0.74)* | 0.96  0.73-1.25 | 0.76  (0.55-1.05) | 0.65  (0.47-0.9)* |
|  | 1.46  (1.13-1.89) | 1.12  (0.86-1.44) | quetiapine | 0.93  0.72-1.2 | 1.56  (1.2-2.03)* | 1.23  (0.89-1.71) | 1.07  (0.78-1.47) |
|  | 1.18  (0.91-1.53) | 0.90  (0.7-1.16) | 0.81  (0.63-1.04) | ziprasidone | 1.68  (1.29-2.18)* | 1.32  (0.96-1.83) | 1.15  (0.84-1.57) |
|  | 1.11  (0.86-1.44) | 0.85  (0.66-1.09) | 0.76  (0.59-0.98) | 0.94  (0.73-1.21) | risperidone | 0.79  (0.57-1.1) | 0.68  (0.5-0.94)* |
|  | 1.06  (0.77-1.46) | 0.81  (0.59-1.11) | 0.72  (0.52-1) | 0.89  (0.65-1.23) | 0.95  (0.69-1.31) | haloperidol | 0.86  (0.6-1.26) |
|  | 1.35  (0.99-1.85) | 1.03  (0.75-1.41) | 0.93  (0.68-1.27) | 1.14  (0.84-1.57) | 1.22  (0.89-1.66) | 1.28  (0.89-1.85) | perphenazine |

*****:P value<0.05 after FDR correction.

Note: The upper figure shows odds ratio (95％ confidence interval) of pairwise comparison of composition ratio between groups with reduction rate of PANSS higher than 50％ and lower than 50％ at week 6. The bottom figure shows odds ratio (95％ confidence interval) of pairwise comparison of composition ratio between high and low trajectory groups of 7 antipsychotic drugs.


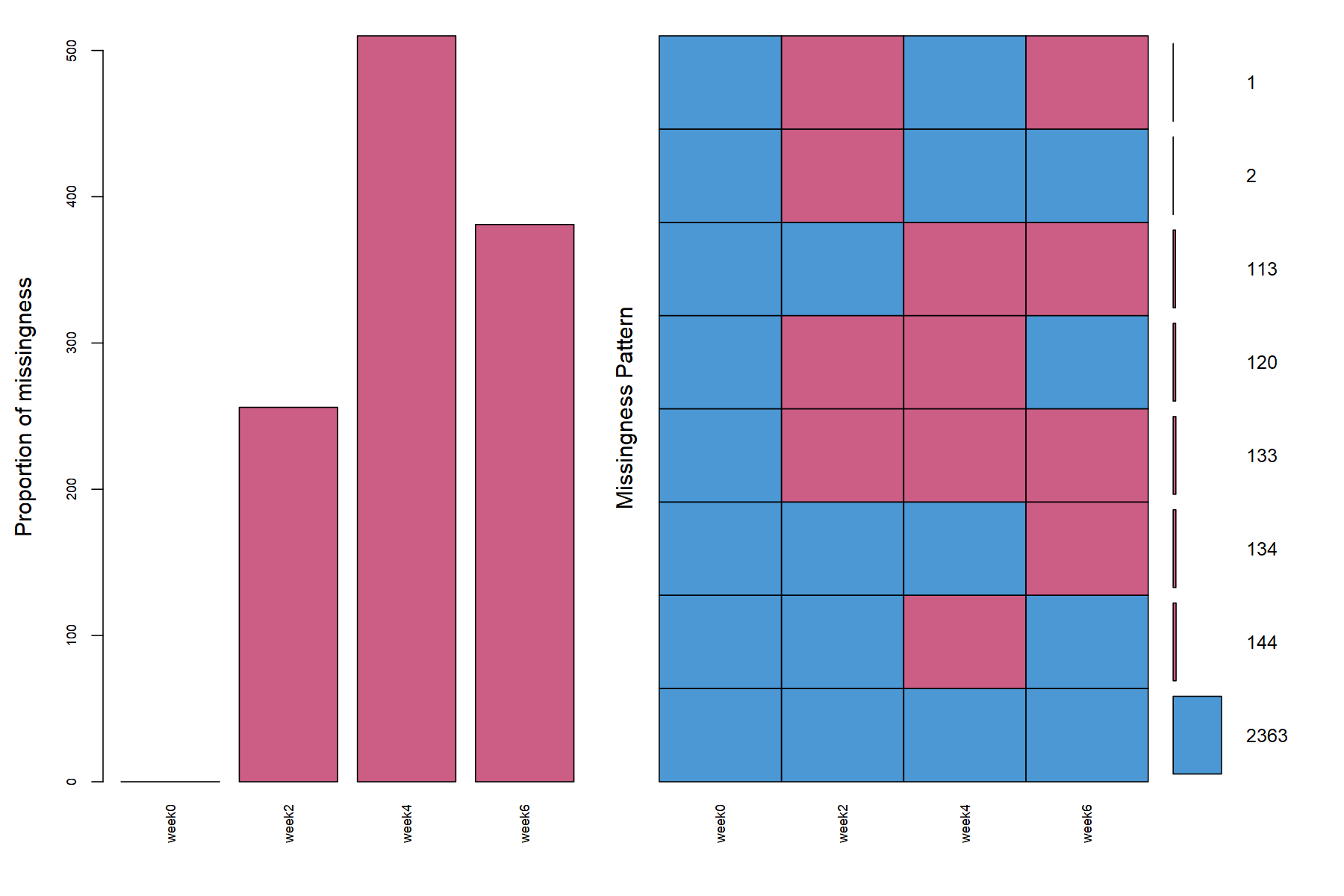


Figure S1: This plot gives numbers to different combinations of missing variables. Blue refers to the observed data and red to the missing data. In the combination plot on the right side, the grid presents all combinations of missing (red) and observed (blue) values present in the data. There are 2363(78.50％) complete observations. The figure on the left shows the missing data each week, with the most missing data in the fourth week.


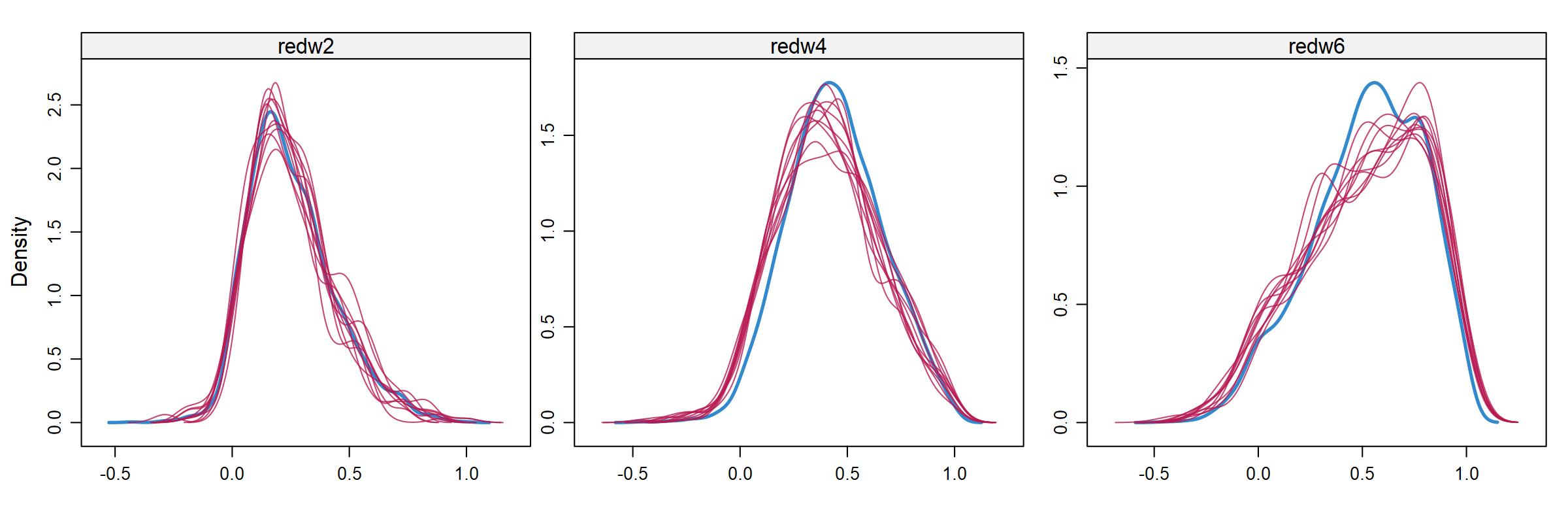


Figure S2: The figure above shows the density curve before and after using multiple imputation in the 2nd, 4th and 6th week. The red curve is the density curve of each multiple imputed datasets, and the blue curve is the density curve of the observed data. Through the t-test, there is no significant difference in the data before and after multiple imputation(p>0.05)


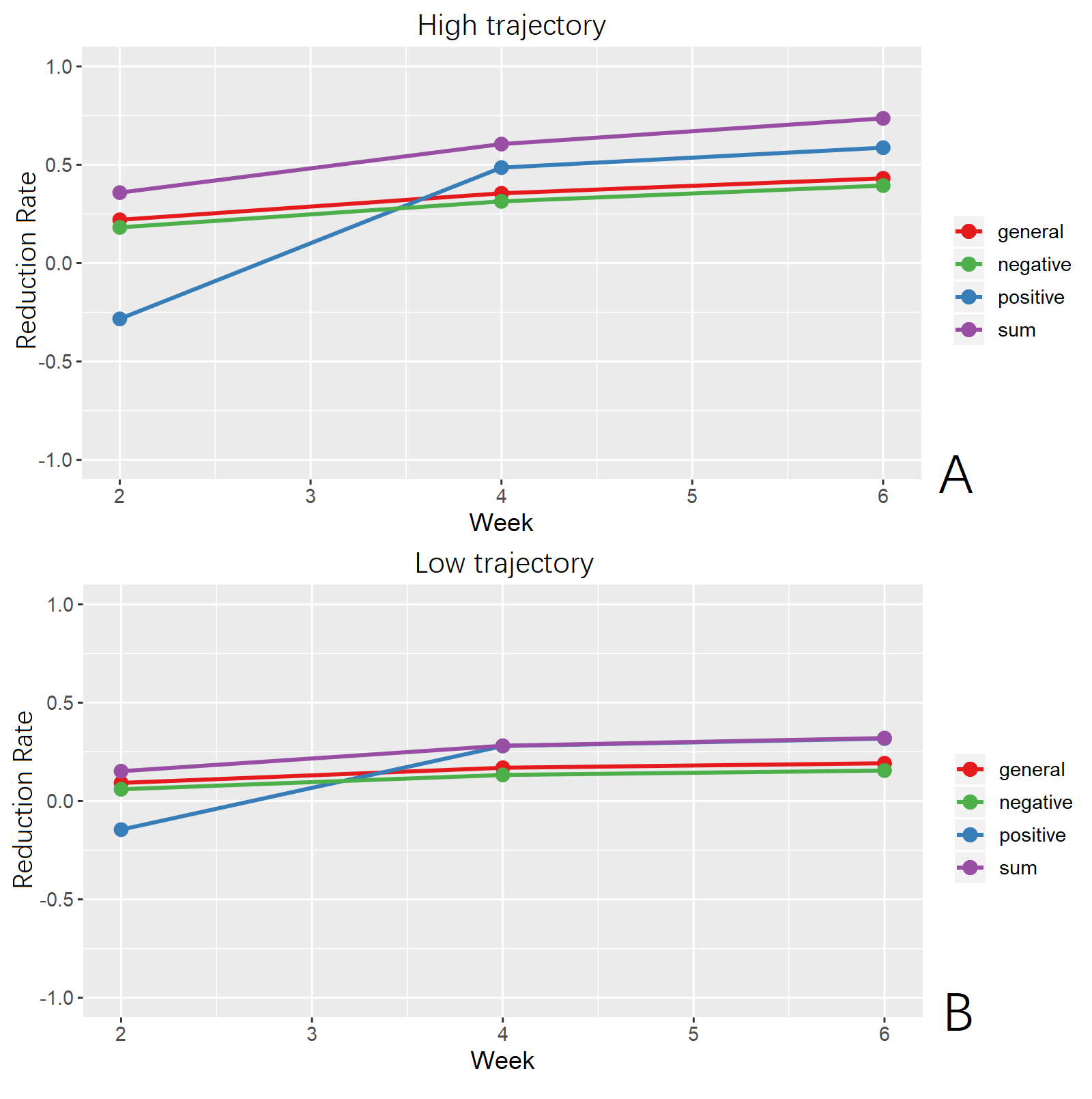


Figure S3 **(A)** The decreasing rate of PANSS total score and each subscale of high trajectory group in the 2nd, 4th and 6th week. **(B)** The decreasing rate of PANSS total score and each subscale of low trajectory group in the 2nd, 4th and 6th week.


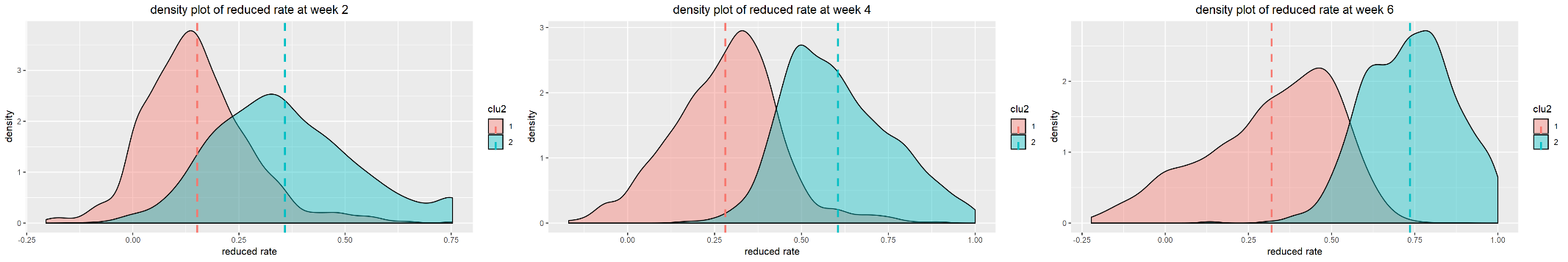


Figure S4 Density plot of reduction rate of PANSS total score of high trajectory group and low trajectory group


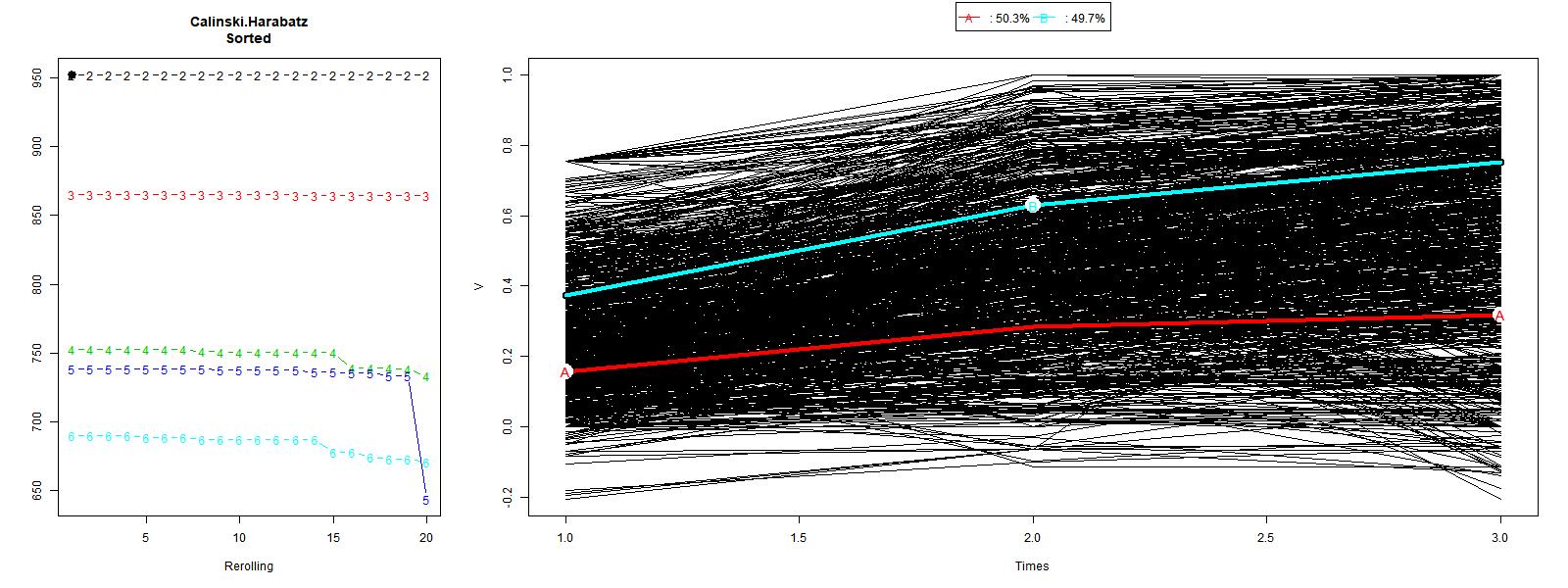


Figure S5 KmL clustering analysis of treatment response (first-episode schizophrenia).


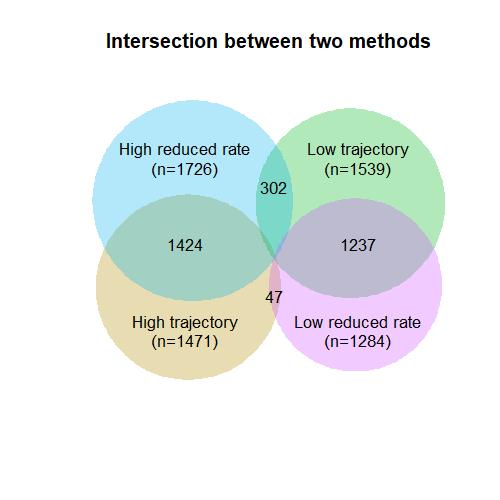


Figure S6: Venn diagram presenting the patients in groups by trajectory analysis and groups by dichotomous thresholds methods. Numbers in the individual sections represent the number of patients. 47 patients were in the high trajectory group ,but the reduction rate of PANSS score in the sixth week was less than 50％. 302 patients were in the low trajectory group，but the reduction rate in the sixth week was more than 50％.


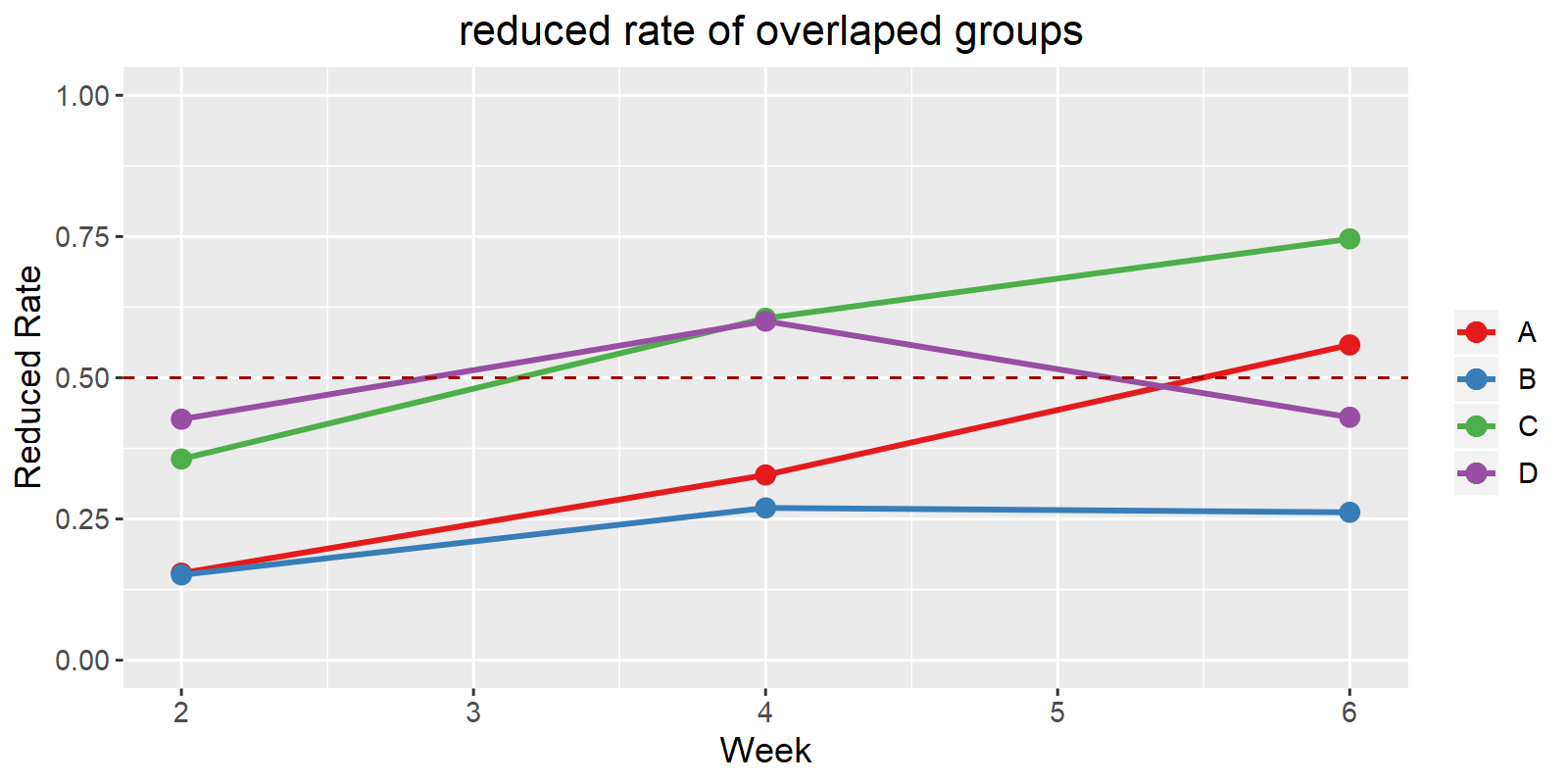


Figure S7 Group A is the total of 302 patients who are in the low trajectory group, but the reduction rate in the sixth week is more than 50％. Group D is patients in the high trajectory group ,but the reduction rate of PANSS score in the sixth week is less than 50％. Group B is in the low-trajectory group and the reduction rate in the sixth week is less than 50, while group C is in the high track group and the reduction rate in the sixth week is more than 50.


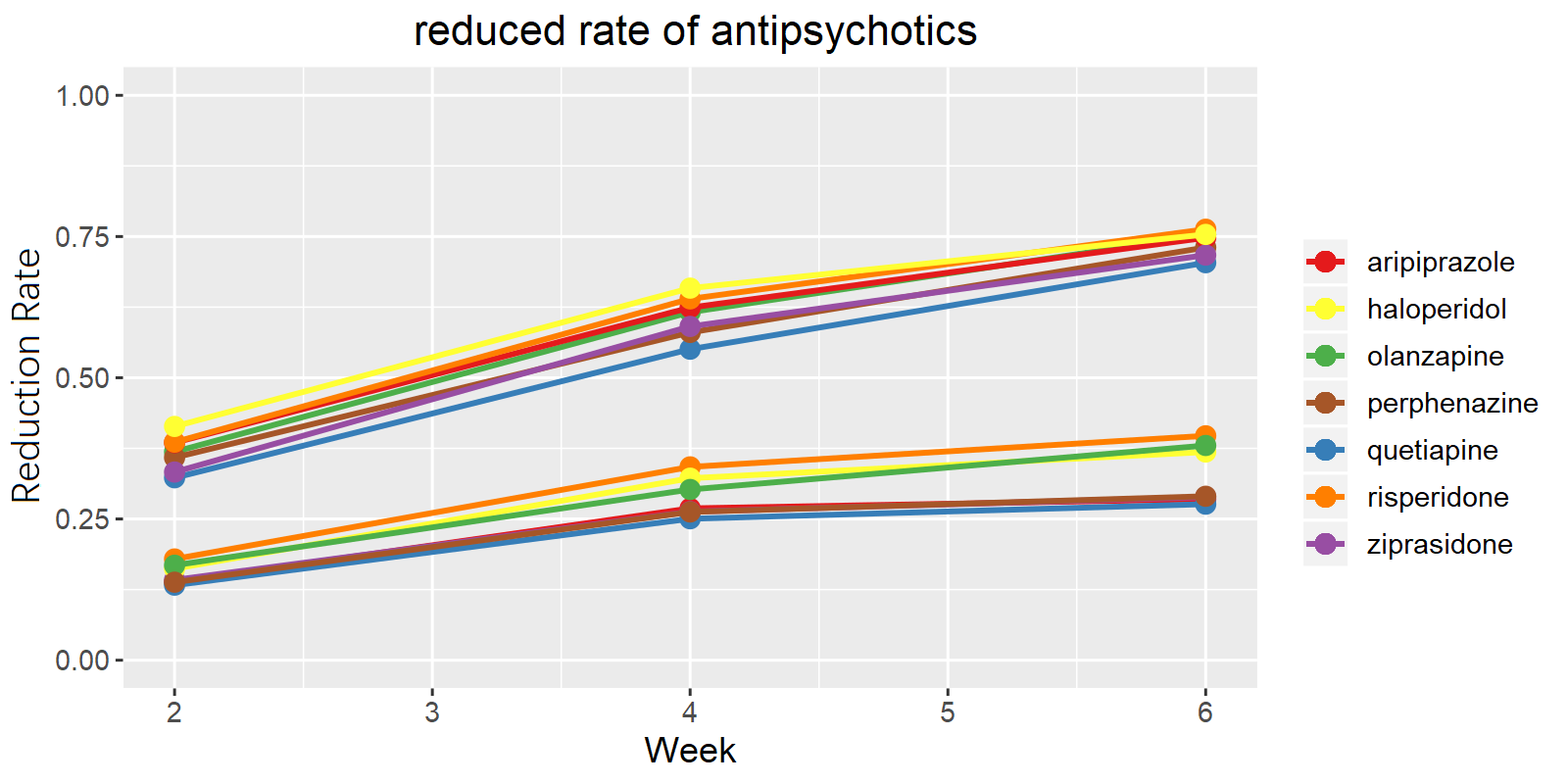


Figure S8：Treatment response trajectories based on the analysis of each antipsychotic medication group separately.


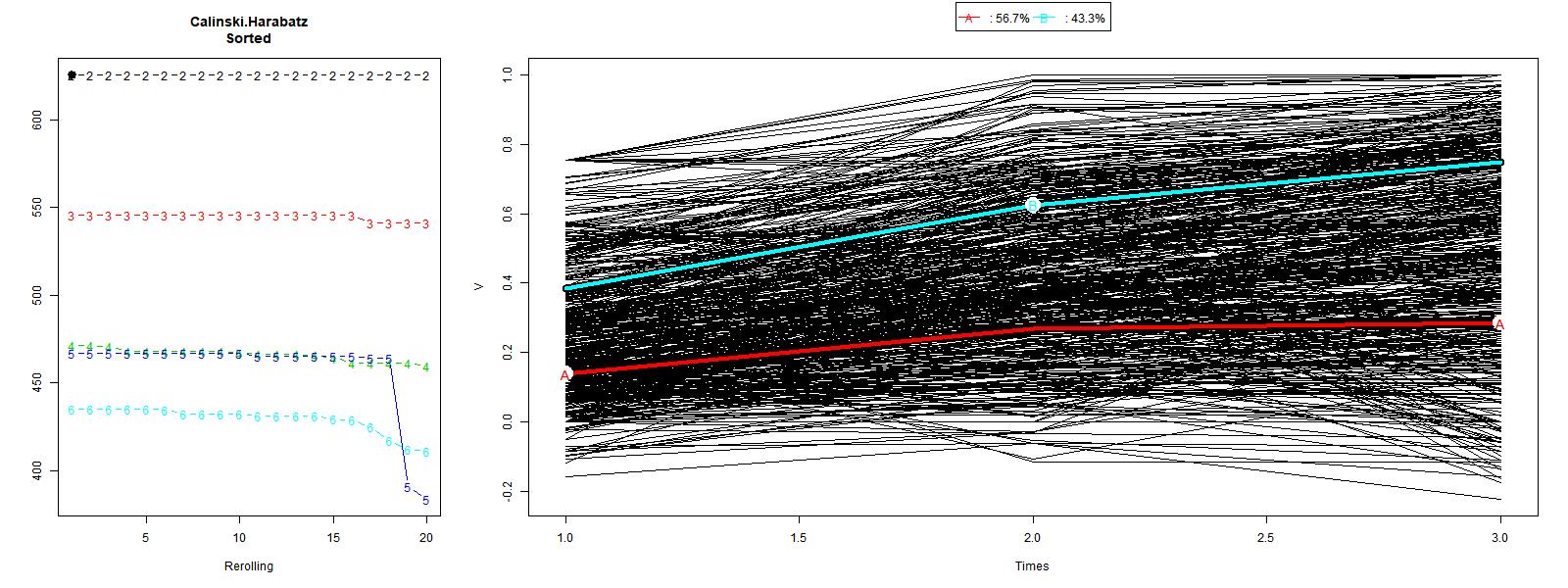


Figure S9 KmL result of Aripiprazole


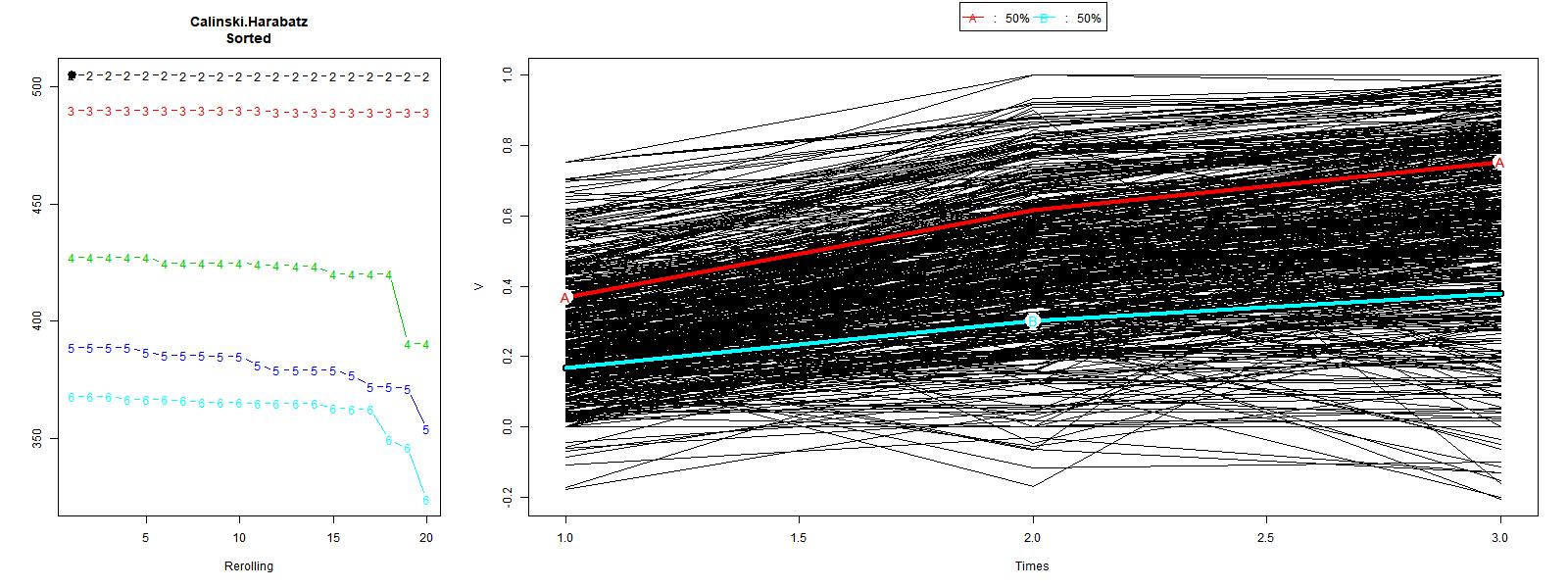


Figure S10 KmL result of Olanzapine


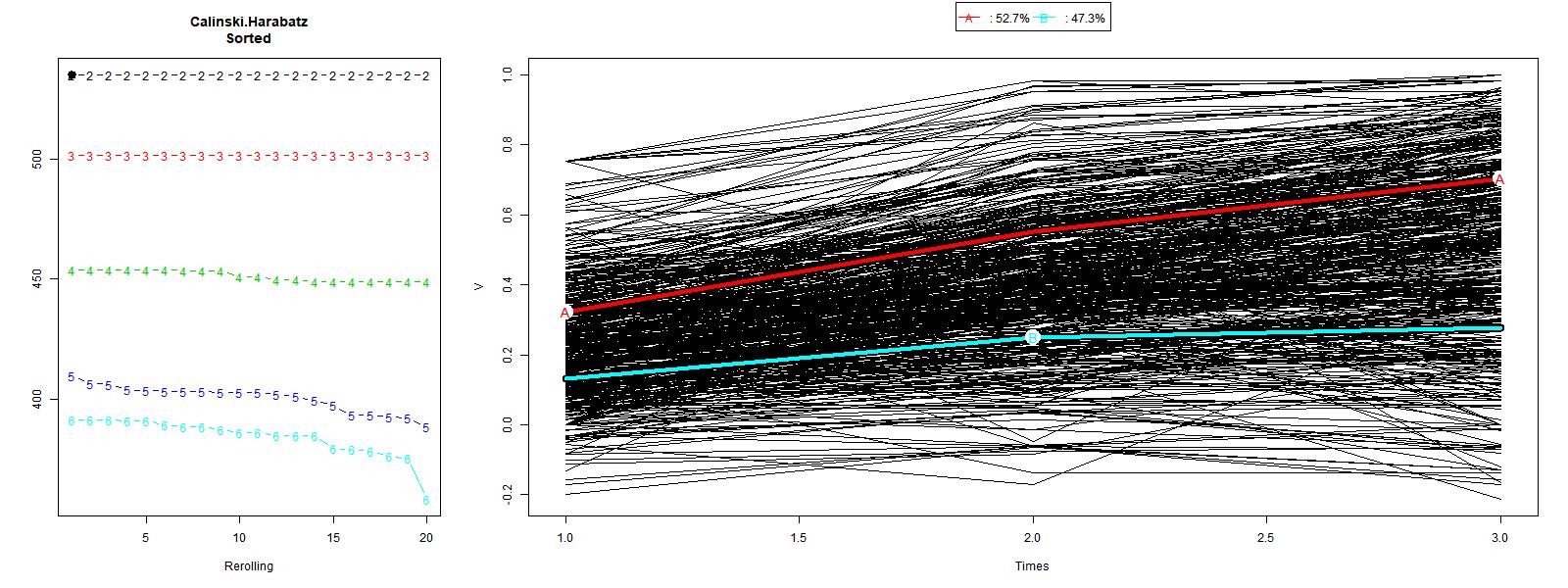


Figure S11 KmL result of Quetiapine


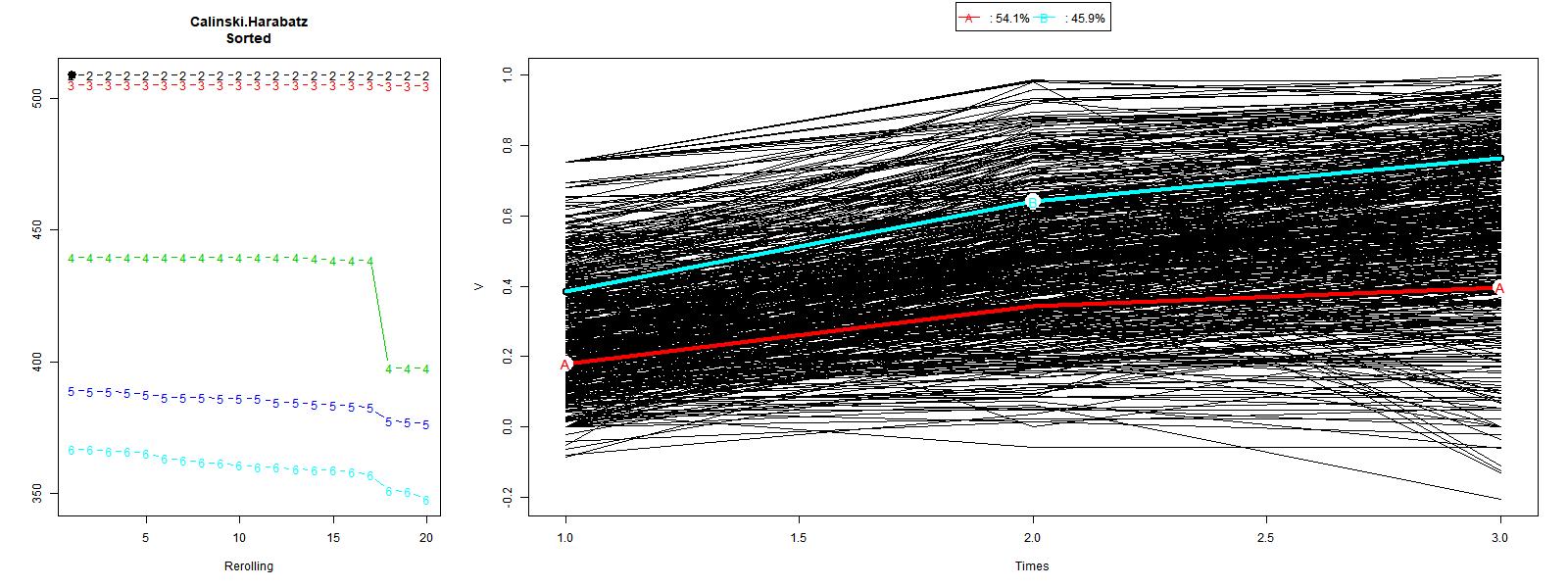


Figure S12 KmL result of Risperidone


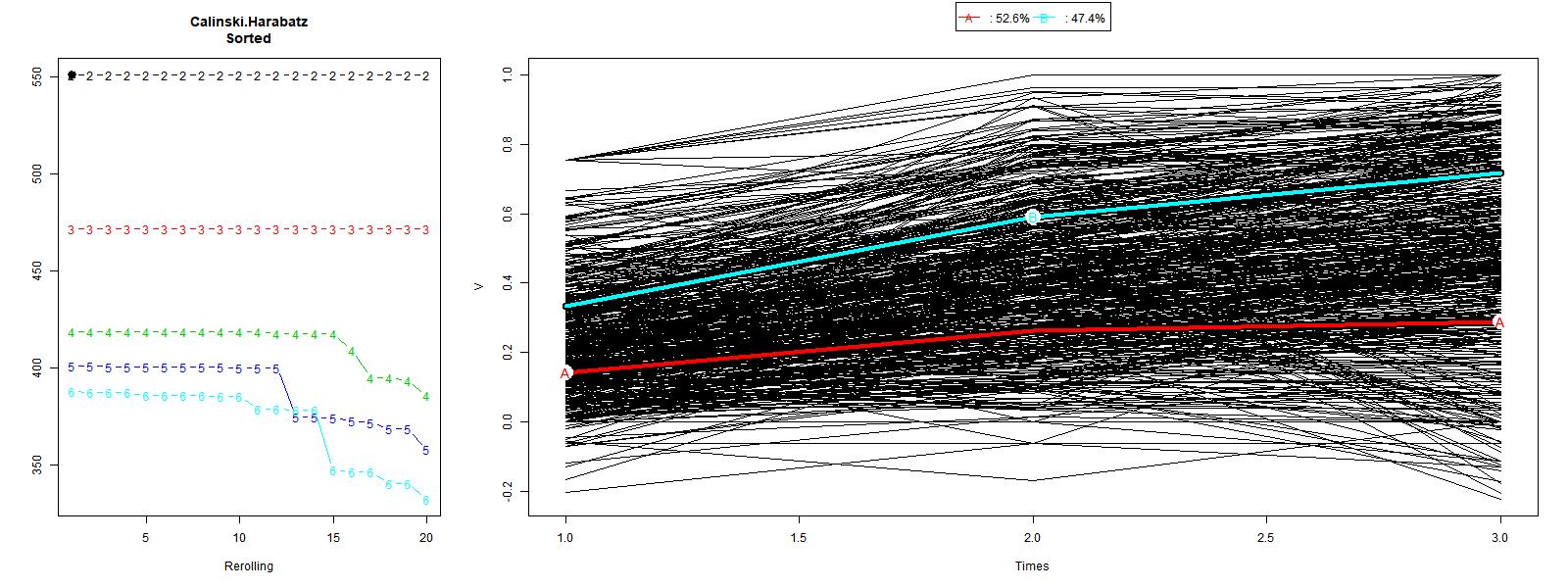


Figure S13 KmL result of Ziprasidone


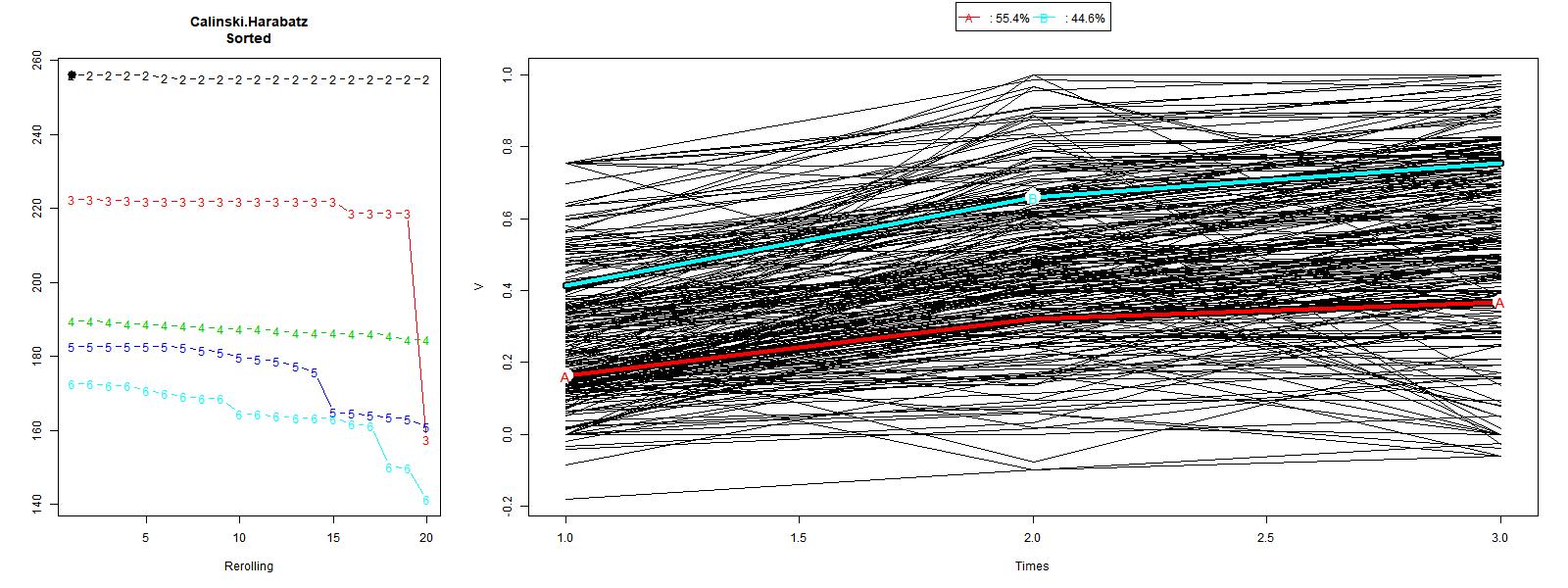


Figure S14 KmL result of Haloperidol


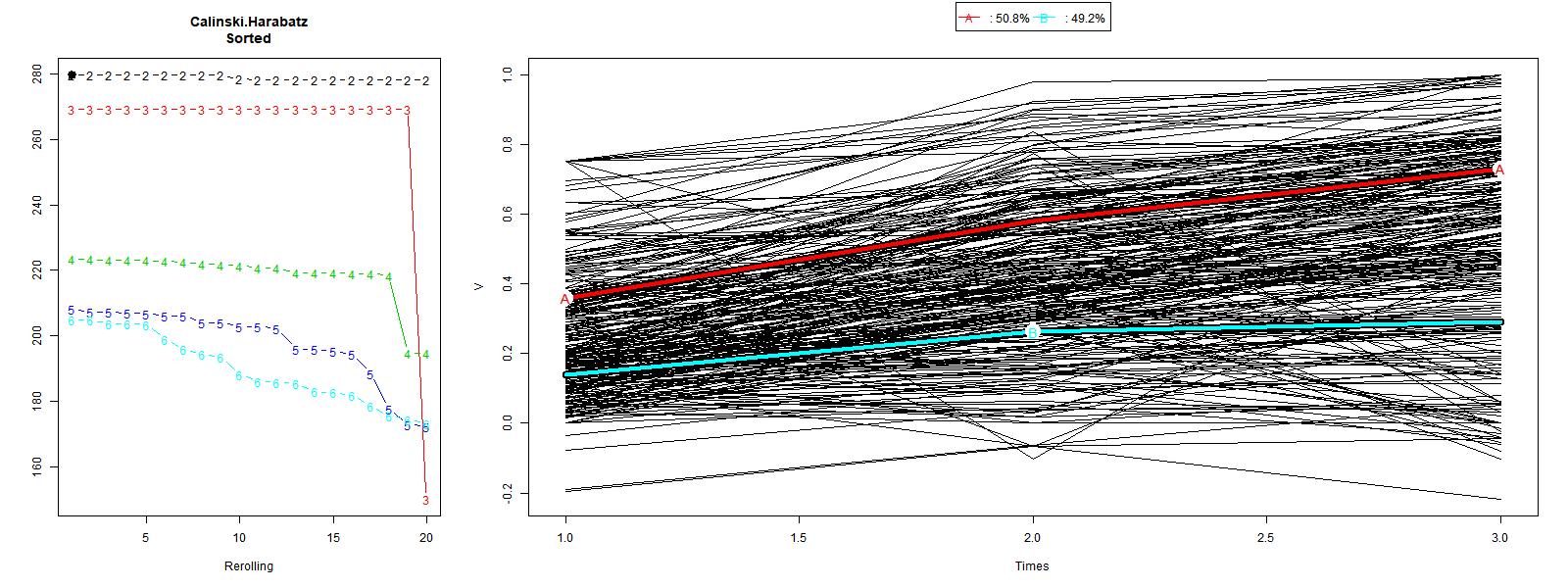


Figure S15 KmL result of Perphenazine
